# Delirium and High Fever Are Associated with Subacute Motor Deterioration in Parkinson Disease: A Nested Case-Control Study

**DOI:** 10.1371/journal.pone.0094944

**Published:** 2014-06-02

**Authors:** Atsushi Umemura, Tomoko Oeda, Satoshi Tomita, Ryutaro Hayashi, Masayuki Kohsaka, Kwiyoung Park, Hiroshi Sugiyama, Hideyuki Sawada

**Affiliations:** 1 Clinical Research Center, National Hospital of Utano, Kyoto, Japan; 2 Department of Neurology, National Hospital of Utano, Kyoto, Japan; Oslo University Hospital, Norway

## Abstract

**Background:**

In Parkinson disease (PD), systemic inflammation caused by respiratory infections such as pneumonia frequently occurs, often resulting in delirium in the advanced stages of this disease. Delirium can lead to cognitive and functional decline, institutionalization, and mortality, especially in the elderly. Inflammation causes rapid worsening of PD motor symptoms and signs, sometimes irreversibly in some, but not all, patients.

**Purpose:**

To identify factors associated with subacute motor deterioration in PD patients with systemic inflammation.

**Methods:**

The association of clinical factors with subacute motor deterioration was analyzed by a case-control study. Subacute motor deterioration was defined as sustained worsening by one or more modified Hoehn and Yahr (H–Y) stages. Using multivariable logistic regression incorporating baseline characteristics (age, sex, PD duration, modified H–Y stage, dementia, and psychosis history) and statistically selected possible predictors (peak body temperature, duration of leukocytosis, and presence of delirium), the odds ratios for these factors were estimated as relative risks.

**Results:**

Of 80 PD patients with systemic inflammation, 26 with associated subacute motor deterioration were designated as cases and the remainder as controls. In the 26 cases, 6 months after its onset the motor deterioration had persisted in 19 patients and resolved in four (three were lost for follow-up). Multivariable logistic regression analysis showed that delirium and body temperature are significantly associated with motor deterioration after systemic inflammation (*P* = 0.001 for delirium and *P* = 0.026 for body temperature), the adjusted odds ratios being 15.89 (95% confidence interval [CI]: 3.23–78.14) and 2.78 (95% CI: 1.13–6.83), respectively.

**Conclusions:**

In patients with PD and systemic inflammation, delirium and high body temperature are strong risk factors for subsequent subacute motor deterioration and such deterioration can persist for over 6 months.

## Introduction

Parkinson disease (PD), a common neurodegenerative disorder, occurs mainly in the elderly. It is characterized by bradykinesia, muscular rigidity, and tremor, these motor symptoms and signs being caused by progressive dopaminergic neuronal degeneration in the brain. Dopaminergic neuronal degeneration progresses insidiously and constantly; accordingly, motor disturbance deteriorates gradually in most patients [1]–[3]. However, some patients experience subacute worsening of motor symptoms in certain situations, the most common of which are infections, anxiety, and medication problems [4], [5]. Most such subacute motor deteriorations are transient and reversible. However, even after adjustment of anti-Parkinsonian medications, they are irreversible in some patients, suggesting that systemic inflammation can be associated with persistent motor exacerbation. Although the mechanism by which systemic inflammation causes subacute worsening of motor symptoms and signs is unknown, evidence obtained in PD animal models in which inflammation is experimentally induced in the central nervous system with attendant systemic inflammatory stimuli has suggested that one possible mechanism is acceleration of dopaminergic neurodegeneration in the substantia nigra [6]–[8].

Delirium, an acute and profound disturbance of consciousness, is accompanied by cognitive and perceptive disturbances and fluctuates daily [9], [10]. Even after recovery from delirium, further declines in cognition [11]–[14] and functional status can occur in the elderly [11], [12], sometimes with consequent institutionalization [11], [14] and mortality [11], [14]. Serious systemic inflammation is a major cause of delirium [15], [16]. Taken together, systemic inflammation with delirium may precipitate subacute deterioration of motor symptoms in PD patients.

The purpose of this study was to identify factors precipitating motor deterioration after systemic inflammation in patients with PD, with the focus on the severity of systemic inflammation and delirium. Using multivariable logistic regression, the odds ratios for the predictors were estimated as relative risks.

## Materials and Methods

### Study Design

A nested case-control study to investigate factors precipitating motor deterioration after systemic inflammation in patients with PD was performed. The odds ratios of motor deterioration according to various precipitating factors were calculated by multivariable logistic regression analysis.

### Study Subjects

The medical records of 888 consecutive hospital patients with PD who fulfilled the United Kingdom Parkinson’s Disease Society Brain Bank clinical diagnostic criteria (steps 1 and 2) [17] and had been admitted to the Department of Neurology of the National Hospital of Utano from November 2006 to April 2013 were reviewed. In accordance with the purpose of the study, eligibility criteria for this study were as follows.

#### Inclusion criteria

(i) Hospital patients with systemic inflammation and (ii) in modified Hoehn and Yahr (H–Y) stage 4 or less at baseline.

#### Exclusion criteria

(i) Delirium within the 6 months prior to the episode of systemic inflammation. (ii) Comorbid conditions such as anoxic encephalopathy, stroke, persistent hypoglycemia, brain surgery, fracture of the legs or vertebrae, myelopathy, and surgery (excluded because these conditions could have interfered with evaluation of modified H–Y stage). (iii) Medication errors during period of evaluation of subacute motor deterioration. (iv) Increased dosages of anti-psychotic drugs or decreased dosages of anti-Parkinsonian medications (L-DOPA equivalent dose [LED] >100 mg/day) in patients with subacute motor deterioration (because worsening could have been induced by such medication changes). (v) Reduction in dosage of anti-psychotic drugs or increase in dosage of anti-Parkinsonian agents (LED >100 mg/day) in patients without subacute motor deterioration (for the same reason). (vi) Missing clinical records.

The study was approved by the Bioethics Committee of Utano National Hospital. According to this committee, informed patient consent was not required because the study was retrospective and the data were analyzed anonymously.

### Definitions of Systemic Inflammation and Subacute Motor Deterioration

The definition of systemic inflammation was a combination of leukocytosis and increased concentration of plasma C-reactive protein (CRP; normal range 0.0–0.5 mg/dL). Leukocytosis was defined as leukocyte or neutrophil count above the normal range (4,000–9,000/µL for leukocytes and 1,480–6,480/µL for neutrophils). Motor deterioration was defined as worsening of modified H–Y stage by one or more stages, this definition being selected for the following three reasons: (i) we assumed that modified H–Y stage would be stable for 6 months; (ii) the study was designed to detect clinically significant exacerbations; and (iii) it was not possible to determine Unified Parkinson’s Disease Rating Scale Part III (UPDRS-III) scores retrospectively.

### Assessed Variables

The following data were collected as possible precipitating factors: variables denoting severity of inflammation (peak values of body temperature, blood leukocyte counts, and plasma CRP concentrations); variable denoting duration of inflammation (duration of leukocytosis); and occurrence of delirium. The duration of leukocytosis was defined as the period from the time of first exceeding the normal range of leukocyte or neutrophil counts to that of their normalization. The highest values for body temperature, leukocyte counts, and CRP concentrations during leukocytosis were identified by assessing body temperature daily and leukocyte counts and CRP concentrations 2–13 times, depending on duration of inflammation, at median intervals of 3 days (IQR: 1–3) in patients without deterioration and 3 days (2–4) in those with deterioration. Delirium was diagnosed retrospectively in accordance with the criteria of the Diagnostic and Statistical Manual of Mental Disorders (DSM-IV) [9] on the basis of notes made in the medical records by physicians and nursing staff. The latter checked patients at 2 h intervals throughout the day and recorded disturbances and fluctuations in consciousness, cognition, and perception.

The baseline characteristics of sex, age of PD onset, age at time of systemic inflammation, PD duration, disease severity (modified H–Y stage and UPDRS-III scores), dementia, Mini-Mental State Examination (MMSE) scores, history of psychosis, initial symptoms (tremor or not), presence of symptomatic orthostatic hypotension, LED, and use of antipsychotic drugs, dopamine agonists, and central anticholinergic drugs (trihexyphenidyl, biperiden, profenamine, piroheptine, and promethazine) were collected. PD duration, UPDRS-III scores, dementia, MMSE scores, presence of symptomatic orthostatic hypotension, and use of dopamine agonists and central anticholinergic drugs were collected from the most recent medical records prior to the onset of systemic inflammation. Diagnoses of dementia were made according to DSM-IV criteria and orthostatic hypotension was diagnosed based on UPDRS-IV criteria. A history of psychosis required either visual or auditory hallucinations or delusions before the onset of systemic inflammation. Modified H–Y stage data were retrospectively collected based on information in the medical records. Modified H–Y stage just before the onset of inflammation was regarded as pre-inflammatory data, and 3 weeks or more after recovery from inflammation as post-inflammatory data. LED was calculated according to the following formula [18]; LED = L-DOPA (mg) (or 1.33×L-DOPA [mg] if taking entacapone) +100× pramipexole (mg) +20×ropinirole (mg) +33×rotigotine (mg) +10×bromocriptine (mg) +0.1×pergolide (µg) +66×cabergoline (mg) +10×selegiline (oral, mg)+amantadine (mg) +10×apomorphine (mg). Those variables were assessed and diagnoses made retrospectively by one of the investigators (AU) based on information in the medical records. Data concerning modified H–Y stage, LED and antipsychotic drugs were collected at the following time points.

#### Evaluation of subacute motor deterioration

These data were obtained at two time points: the most recent records before onset and after recovery from systemic inflammation. The median interval was 56 days (range: 28–135) for patients without serious deterioration and 66 days (range: 23–133) for those with such deterioration.

**Evaluation of progression of motor symptoms before systematic inflammation.** These data were collected from 3 to 12 months before the baseline assessment (median interval: 188 days [range: 124–336] for non-deteriorated patients, 189 days [range: 133–326] for deteriorated). The records of 11 patients were unavailable because they had been evaluated at other hospitals.

Each variable was treated as follows: body temperature, leukocyte count, CRP concentration, age of PD onset, age at time of systemic inflammation, PD duration, UPDRS-III score, MMSE score, and LED as scale variables; duration of leukocytosis (1–3 days, 4–8 days, or 9–34 days) and modified H–Y (stage 2, 2.5–3, or 4) as ordinal variables; delirium, sex, dementia, history of psychosis, initial symptoms, presence of symptomatic orthostatic hypotension, and use of dopamine agonists and central anticholinergic drugs as dichotomous nominal variables.

### Statistical Analysis

Because of its non-Gaussian distribution, scale variables were compared between cases and controls by nonparametric Mann–Whitney U test. Ordinal variables and nominal variables were statistically analyzed using the X^2^ test or Fisher’s exact test, respectively.

Associations between possible precipitating factors and motor deterioration after systemic inflammation were statistically analyzed by multivariable logistic regression. First, multicollinearity between scale variables was examined by Spearman’s rank correlation coefficient, following which age of PD onset was excluded from multivariable logistic regression analysis because of multicollinearity with age (Spearman’s rank correlation coefficient = 0.81, *P*<0.001). Baseline characteristics, including sex, age, PD duration, modified H–Y stage, dementia, and history of psychosis, were incorporated into the statistical model. Precipitating factors, including body temperature, duration of leukocytosis, and delirium, were statistically selected by a likelihood test (backward). The fitness of the model was statistically analyzed using the Hosmer–Lemeshow test and predictive accuracy. All statistical analyses were carried out using GraphPad Prism for Windows ver. 5.0 (GraphPad Software, San Diego, CA, USA, http://www.graphpad.com) and the statistical software program SPSS 18.0 (PASW statistics, http://www.spss.com/). *P* values of <0.05 were considered statistically significant.

## Results

### Characteristics of Subjects

**Figure 1:**
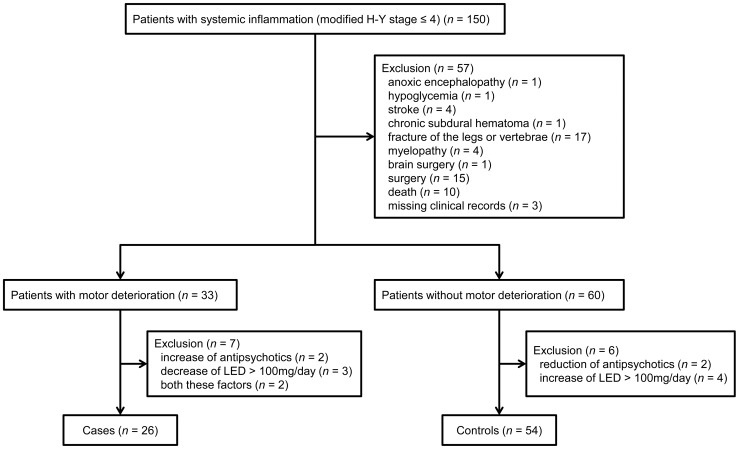
Flow diagram showing selection of eligible patients and the enrollment process. H–Y, Hoehn and Yahr; LED, L-DOPA equivalent dose.

Of the 150 eligible patients, 33 had motor deterioration after inflammation and 60 no deterioration; the remaining 57 patients were excluded (54 because of concomitant disease, including death [*n* = 10], affecting modified H–Y stage and three because of missing data). Changes in medication dosage accounted for exclusion of a further seven patients with, and six without, motor deterioration. Of the remaining 80 patients, 26 with motor deterioration after systemic inflammation were designated as cases and the remaining 54 as controls (Figure 1). Of the 80 study participants, 47 had been admitted because of inflammation whereas the remaining 33 had developed inflammation during hospitalization. There were no significant differences between these groups in the interval between baseline data in medical records and onset of systemic inflammation and causes of inflammation; however, malignant syndrome tended to be more often responsible in the former and pyelonephritis and prostatitis in the latter (Table S1). Median length of hospital stay was 36.0 days (IQR: 10.0–94.0) for controls and 168.5 days (68.8–319.5) for cases. Forty-six of 54 controls and 15 of 26 cases were followed for over 365 days after recovery from systemic inflammation; the remainder were followed up for a median (IQR) of 263.0 days (91.0–292.0) for controls and 238.0 days (155.0–309.0) for cases; most of the latter patients were censored because they were transferred (57.9%, 11 of 19 patients). Modified H–Y stage changed as follows.

#### Subacute motor deterioration after systemic inflammation

Of 26 cases, 22 exacerbated by one grade (range: 1–2.5). Two control patients changed by 0.5 of a grade. All patients were appropriately medicated; in four cases anti-Parkinsonian drug dosage was increased (LED range: 17–300 mg/day) and in eight controls it was decreased (LED range: 67–361 mg/day).

In the current study, exacerbations of motor function attributable to inflammation were detected by identifying worsening of modified H–Y stages between the following two time points: before inflammation and after recovery from inflammation. Because modified H–Y stage may have worsened for reasons other than inflammation between these two time points, changes in modified H–Y during the period before onset of inflammation were assessed in 69 patients for whom sufficient data were available. Of these 69 patients, six had progressed by 0.5 of a grade (one case, five controls); modified H–Y stage was stable in the remaining 63 patients. Four patients had reductions in anti-psychotic drug dosage and three increases in dosage of anti-Parkinsonian agents (LED >100 mg/day, range: 150–183 mg/day) (Table S2).

Relevant clinical and other patient variables are shown in Table 1. Of the variables denoting severity of inflammation, there was a significant difference between cases and controls in body temperature (*P*<0.001). The difference between cases and controls in duration of leukocytosis was highly significant (*P*<0.001). Leukocytosis continued for 9 days or longer in half the cases and, in contrast, in only 1.9% of control patients. Delirium occurred in 10 control patients (18.5%) and 21 cases (80.8%); this difference is highly significant (*P*<0.001). There was a history of psychosis in 84.6% of cases and 59.3% of controls (*P* = 0.040). There were no significant differences in other baseline characteristics between cases and controls. The most common cause of systemic inflammation was pneumonia in both cases and controls; there was no significant difference between the groups in cause of inflammation (Table 2). Of the 26 cases with motor deterioration, 6 months later the motor deterioration had persisted in 19 patients and resolved in four; relevant records were unavailable for the remaining three patients. We found no significant differences in any factors between patients who did and did not recover from their motor deterioration (Table S3).

**Table 1:** Relevant clinical and other variables of study participants.

		Controls (n = 54)	Cases (n = 26)	*P*
Male, n [%]		33 [61.1]	20 [76.9]	0.210
Age of PD onset, mean [SD] Y		65.2 [9.8]	67.1 [10.4]	0.334
Age (at time of systemic inflammation), mean [SD] Y		75.8 [7.0]	76.0 [9.1]	0.471
PD duration, mean [SD] Y		10.7 [6.4]	8.9 [3.5]	0.431
Modified H–Y stage, n [%]	2	7 [13.0]	0 [0.0]	0.151
	2.5–3	27 [50.0]	14 [53.8]	
	4	20 [37.0]	12 [46.2]	
UPDRS-III score, mean [SD], n		26.5 [12.4], 8	35.4 [9.2], 8	0.169
Dementia, n [%]		29 [53.7]	17 [65.4]	0.347
MMSE score, mean [SD], n		24.3 [4.3], 17	20.8 [6.0], 8	0.160
History of psychosis, n [%]		32 [59.3]	22 [84.6]	0.040
Initial symptoms, non-tremor n [%]		36 [66.7]	22 [84.6]	0.114
Symptomatic orthostatic hypotension, n [%]		12 [22.2]	10 [38.5]	0.181
LED, median [range] mg/day		600 [0–1673]	481 [300–898]	0.164
Use of dopamine agonists, n [%]		20 [37.0]	4 [15.4]	0.068
Use of central anticholinergic drugs, n [%]		4 [7.4]	1 [3.8]	1.000
Delirium, n [%]		10 [18.5]	21 [80.8]	<0.001
Peak body temperature, mean [SD]°C		37.8 [0.8]	38.7 [0.8]	<0.001
Peak leukocyte count, mean [SD]×10^3^/µl		11.6 [2.9]	12.4 [3.6]	0.345
Peak plasma CRP concentration, mean [SD] mg/dl		8.5 [5.9]	11.5 [6.9]	0.057
Duration of leukocytosis, n [%]	1–3 days	31 [58.5]	7 [26.9]	<0.001
	4–8 days	21 [39.6]	6 [23.1]	
	9–34 days	1 [1.9]	13 [50.0]	

**Table 2:** Causes of systemic inflammation.

	Controls (n = 54)	Cases (n = 26)
Pneumonia, n [%]	28 [51.9]	14 [53.8]
Upper respiratory inflammation, n [%]	5 [9.3]	1 [3.8]
Influenza, n [%]	0 [0.0]	1 [3.8]
Pyelonephritis, n [%]	2 [3.7]	3 [11.5]
Prostatitis, n [%]	5 [9.3]	1 [3.8]
Gastroenteritis, n [%]	3 [5.6]	0 [0.0]
Cholecystitis, n [%]	2 [3.7]	1 [3.8]
Cellulitis, n [%]	3 [5.6]	2 [7.7]
Malignant syndrome, n [%]	3 [5.6]	1 [3.8]
Gouty attack, n [%]	1 [1.9]	0 [0.0]
Burn, n [%]	0 [0.0]	1 [3.8]
Not available, n [%]	2 [3.7]	1 [3.8]

### Adjusted Odds Ratios for Motor Deterioration

Multivariable logistic regression analysis showed that delirium and body temperature were significantly associated with motor deterioration after systemic inflammation (*P* = 0.001 for delirium and *P* = 0.026 for body temperature). The adjusted odds ratios were 15.89 for delirium (95% CI: 3.23–78.14) and 2.78 for body temperature (95% CI: 1.13–6.83) (Table 3). The Hosmer–Lemeshow test showed no significance (*P* = 0.712) and predictive accuracy was 89.7%.

**Table 3:** Adjusted odds ratios for motor deterioration (adjusted for age, sex, PD duration, modified H–Y stage, dementia, and history of psychosis).

	B	Odds ratio [95% CI]	*P*
Delirium (yes *vs.* no)	2.77	15.89 [3.23–78.14]	0.001
Peak body temperature (per one°C)	1.02	2.78 [1.13–6.83]	0.026
Sex (male *vs.* female)	1.10	3.01 [0.48–18.77]	0.238
Age (at time of systemic inflammation) (per one year)	−0.06	0.94 [0.85–1.05]	0.256
PD duration (per one year)	−0.20	0.82 [0.69–0.97]	0.019
Modified H–Y stage (4 *vs.* 2.5–3 *vs.* 2)	0.98	2.67 [0.52–13.88]	0.242
Dementia (yes *vs.* no)	−1.05	0.35 [0.06–2.16]	0.259
History of psychosis (yes *vs.* no)	2.19	8.97 [0.89–90.83]	0.063

## Discussion

In this study, we found that delirium was significantly associated with subacute motor deterioration in PD patients after inflammation (Table 2). Although body temperature was also associated with subacute motor deterioration, the association of delirium with motor deterioration was significant even after adjustment for body temperature. Medication error, often reversible, is another reason for motor deterioration [4], [5]; however, our study design excluded the effects of medication factors. Of 23 patients who deteriorated because of systemic inflammation, the changes were irreversible in 19 for whom we had sufficient data, even 6 months after recovery from inflammation. There were no significant differences between patients with transient deterioration and those with persistent deterioration in any of the assessed factors, including medications (Table S3). Multivariate logistic regression analysis of a subgroup excluding patients with transient deterioration produced similar results (Table S4). We diagnosed delirium according to notes made in the medical records by physicians and nursing staff. When delirium is diagnosed based on recording by nurses, the sensitivity is reportedly relatively low and the specificity high [19]; therefore, mild delirium may have been overlooked in this study, in which the prevalence of delirium was 38.8% (31 of 80 patients comprising 10 of 34 non-demented [29.4%] and 21 of 46 demented patients [45.7%]). This is in line with previous reports, the prevalence having been reported as 5–30% of all elderly people admitted to emergency departments [20]–[22], 10–20% of non-demented patients with PD [23], [24], and 46% of demented PD patients [24]. Anticholinergic drugs can also induce psychosis [25] and delirium [24], [26] in PD patients. Only 6.3% (5/80 patients) received central anticholinergic drugs: too few to allow assessment of any association with these drugs.

As shown in Table 2, delirium during inflammation was the strongest precipitator of subacute motor deterioration. In animal PD models, cytokine activation caused by systemic inflammatory stimuli induces degeneration of nigrostriatal dopaminergic neurons [6]–[8]. In the brain, microglia and cytokines play an essential role in delirium [27]–[29]. Although the pathophysiology underlying subacute motor deterioration remains unclear, we postulate that inflammation involving the central nervous system leads to cytokine activation that could both elicit delirium and cause dopaminergic neurodegeneration resulting in motor deterioration. In this study, we found that body temperature was the second strongest precipitator of motor deterioration (Table 2). Oxidative stress adversely affects dopaminergic neurons in PD patients [30] as well as in animal and cell culture PD models, leading to dopaminergic neurodegeneration in the presence of neuroinflammatory processes [31], [32]. These data suggest that dopaminergic neurons might be susceptible to serious systemic inflammation, raising the possibility that early treatment of inflammation could prevent motor deterioration.

Changes in UPDRS-III score can affect quality of life even when modified H–Y stage is stable [33]. However, in this retrospective study, it was not possible to accurately determine UPDRS-III scores based on data in the medical records. Because we used the more robust but less sensitive measure of modified H–Y stage to assess the course of PD in our study patients, we were unable to assess any associations between mild motor deterioration and various clinical factors. However, we did identify some precipitators of motor deterioration, including medication errors and comorbidities such as delirium, according to the study aim. Although our inability to identify precipitators of mild motor deterioration is a limitation of this study, we believe the precipitators we did establish are important for neurologists treating patients with PD.

In summary, our study provides further evidence concerning predictors of motor deterioration after systemic inflammation in patients with PD. The present data indicate that delirium has the strongest association.

## Supporting Information

Table S1 **Comparison between patients who were admitted because of inflammation and those who developed inflammation during hospitalization.**
(PDF)

Table S2 **Progression of motor symptoms in the period before onset of systemic inflammation.**
(PDF)

Table S3 **Relevant clinical and other data of patients with both persistent and transient deterioration.**
(PDF)

Table S4 **Adjusted odds ratios for persistent motor deterioration (adjusted for age, sex, PD duration, modified H–Y stage, dementia, and history of psychosis).**
(PDF)
