# Supplementary material for: Delirium and High Fever Are Associated with Subacute Motor Deterioration in Parkinson Disease: A Nested Case-Control Study
Source: PLoS One. 2014 Jun 2;9(6):e94944. doi: 10.1371/journal.pone.0094944 (PMC4041721; doi:10.1371/journal.pone.0094944)
Supplement: Table S1 — Comparison between patients who were admitted because of inflammation and those who developed inflammation during hospitalization. (PDF) [file pone.0094944.s001.pdf]

Table S1. Comparison between patients who were admitted because of inflammation and those who developed inflammation during hospitalization.

		Patients with inflammation on admission (n=47)	Patients who developed inflammation during hospitalization (n=33)
Interval between baseline data in medical records and onset of systemic inflammation, median [range] days		21 [2-61]	21 [1-112]
Causes of systemic inflammation	Pneumonia, n [%]	25 [53.2]	17 [51.5]
	Upper respiratory inflammation, n [%]	3 [6.4]	3 [9.1]
	Influenza, n [%]	0 [0.0]	1 [3.0]
	Pyelonephritis, n [%]	1 [2.1]	4 [12.1]
	Prostatitis, n [%]	2 [4.3]	4 [12.1]
	Gastroenteritis, n [%]	3 [6.4]	0 [0.0]
	Cholecystitis, n [%]	3 [6.4]	0 [0.0]
	Cellulitis, n [%]	4 [8.5]	1 [3.0]
	Malignant syndrome, n [%]	4 [8.5]	0 [0.0]
	Gouty attack, n [%]	0 [0.0]	1 [3.0]
	Burn, n [%]	1 [2.1]	0 [0.0]
	Not available, n [%]	1 [2.1]	2 [6.1]
