# Supplementary material for: Delirium and High Fever Are Associated with Subacute Motor Deterioration in Parkinson Disease: A Nested Case-Control Study
Source: PLoS One. 2014 Jun 2;9(6):e94944. doi: 10.1371/journal.pone.0094944 (PMC4041721; doi:10.1371/journal.pone.0094944)
Supplement: Table S2 — Progression of motor symptoms in the period before onset of systemic inflammation. (PDF) [file pone.0094944.s002.pdf]

Table S2. Progression of motor symptoms in the period before onset of systemic inflammation.

		Controls (n=49)	Cases (n=20)
Interval, median [range] days		188 [124–336]	189 [133–326]
Change in modified H-Y stage, n [%]	No change	44 [89.8]	19 [95.0]
	Worsened by 0.5	5 [10.2]	1 [5.0]
