# Supplementary material for: Delirium and High Fever Are Associated with Subacute Motor Deterioration in Parkinson Disease: A Nested Case-Control Study
Source: PLoS One. 2014 Jun 2;9(6):e94944. doi: 10.1371/journal.pone.0094944 (PMC4041721; doi:10.1371/journal.pone.0094944)
Supplement: Table S3 — Relevant clinical and other data of patients with both persistent and transient deterioration. (PDF) [file pone.0094944.s003.pdf]

Table S3. Relevant clinical and other data of patients with both persistent and transient deterioration.

		Cases (n=23)		
		Persistent deterioration (n=19)	Transient deterioration (n=4)	*P*
Male, n [%]		14 [73.7]	3 [75.0]	1.000
Age of PD onset, mean [SD] Y		68.7 [9.7]	67.5 [7.3]	0.871
Age (at time of systemic inflammation), mean [SD] Y		77.7 [7.8]	76.8 [7.9]	0.968
PD duration, mean [SD] Y		9.1 [4.0]	9.3 [1.0]	1.000
Modified H-Y stage, n [%]	2.5–3	10 [52.6]	2 [50.0]	0.924
	4	9 [47.4]	2 [50.0]	
UPDRS-III score, mean [SD], n		34.6 [9.6], 7	41, 1	NA
Dementia, n [%]		12 [63.2]	3 [75.0]	1.000
MMSE score, mean [SD], n		19.8 [5.7], 5	26.0 [5.7], 2	0.381
History of psychosis, n [%]		16 [84.2]	4 [100.0]	1.000
Initial symptoms, non-tremor n [%]		18 [94.7]	3 [75.0]	0.324
Symptomatic orthostatic hypotension, n [%]		6 [31.6]	2 [50.0]	0.589
LED, median [range] mg/day		500 [300–898]	624 [400–700]	0.655
Use of dopamine agonists, n [%]		3 [15.8]	0 [0.0]	1.000
Use of central anticholinergic drugs, n [%]		1 [0.5]	0 [0.0]	1.000
Delirium, n [%]		15 [78.9]	4 [100.0]	1.000
Peak body temperature, mean [SD] °C		38.6 [0.8]	38.9 [0.4]	0.701
Peak leukocyte count, mean [SD] $\times 10^3/\mu l$		12.5 [4.1]	12.8 [2.4]	0.490
Peak plasma CRP concentration, mean [SD] mg/dl		11.7 [7.4]	12.6 [6.7]	0.766
Duration of leukocytosis, n [%]	1–3 days	4 [21.1]	1 [25.0]	0.959
	4–8 days	4 [21.1]	1 [25.0]	
	9–34 days	11 [57.9]	2 [50.0]	
