# Supplementary material for: Delirium and High Fever Are Associated with Subacute Motor Deterioration in Parkinson Disease: A Nested Case-Control Study
Source: PLoS One. 2014 Jun 2;9(6):e94944. doi: 10.1371/journal.pone.0094944 (PMC4041721; doi:10.1371/journal.pone.0094944)
Supplement: Table S4 — Adjusted odds ratios for persistent motor deterioration (adjusted for age, sex, PD duration, modified H–Y stage, dementia, and history of psychosis). (PDF) [file pone.0094944.s004.pdf]

Table S4. Adjusted odds ratios for persistent motor deterioration (adjusted for age, sex, PD duration, modified H-Y stage, dementia and history of psychosis).

	B	Odds ratio [95% CI]	*P*
Delirium (yes *vs.* no)	2.36	10.60 [2.06–54.51]	0.005
Peak body temperature (per one °C)	1.02	2.78 [1.10–7.03]	0.032
Sex (male *vs.* female)	1.06	2.88 [0.46–18.14]	0.259
Age (at time of systemic inflammation) (per one year)	- 0.05	0.95 [0.85–1.07]	0.390
PD duration (per one year)	- 0.19	0.83 [0.70–0.98]	0.028
Modified H-Y stage (4 *vs.* 2.5–3 *vs.* 2)	1.06	2.88 [0.51–16.29]	0.231
Dementia (yes *vs.* no)	- 0.95	0.39 [0.06–2.42]	0.308
History of psychosis (yes *vs.* no)	2.25	9.44 [0.81–110.04]	0.073
